# PURPOSE IN LIFE, PHYSICAL ACTIVITY AND FUTURE THOUGHTS: RESULTS FROM DAILY LIFE ASSESSMENT IN YOUNG AND OLDER ADULTS

**DOI:** 10.1093/geroni/igae098.3696

**Published:** 2024-12-31

**Authors:** Nathan Lewis, Yoonseok Choi, Jennifer Lay, Peter Graf, Da Jiang, Atiya Mahmood, Helene H Fung, Christiane Hoppmann

**Affiliations:** University of British Columbia, Vancouver, British Columbia, Canada; University of British Columbia, Vancouver, British Columbia, Canada; University of Exeter, Exeter, England, United Kingdom; University of British Columbia, Vancouver, British Columbia, Canada; Education University of Hong Kong, Hong Kong, China (People’s Republic); Simon Fraser University, Vancouver, British Columbia, Canada; The Chinese University of Hong Kong, Hong Kong, China (People’s Republic); The Chinese University of Hong Kong, Hong Kong, China (People’s Republic)

## Abstract

Theoretical work suggests a sense of purpose in life may encourage health-promoting behaviors by facilitating a more future-oriented time perspective about one’s everyday goals and actions. However, little research has investigated whether people with a strong sense of purpose are thinking more about the future in their everyday lives and whether this changes across adulthood. Using ecological momentary assessment (EMA), the present study aimed to investigate associations between a sense of purpose in life, age, thoughts about the future, and physical activity. A total of 256 younger and older adults living in Canada and Hong Kong (Mage=48.0, SD=24.6, range=18-85, 68% women) completed electronic assessments 3x/day for 10 days, recording their current thoughts and how much these were directed towards the future. A subset of participants also completed a 6-month follow-up where they reported their physical activity over the past week. Multilevel models indicated those with a higher in sense of purpose reported more future-oriented thoughts across the EMA period, adjusting for self-rated health and demographics (b=3.12, 95% CI=0.33–5.90, p=.028). Associations between sense of purpose and thoughts of the future were consistent across younger and older adults. While a sense of purpose was associated with more future-oriented thoughts, these thoughts did not predict subsequent physical activity 6 months later (b=-0.00, p=.998). Findings support the notion that adults higher in purpose direct their thoughts more towards the future in daily life, yet more work is needed to uncover if and how this impacts health behavior decisions.

